# Trust: The Missing Dimension in the Food Retail Transition in Thailand

**DOI:** 10.1080/00664677.2016.1174101

**Published:** 2016-06-08

**Authors:** C. Banwell, M. Kelly, J. Dixon, S-A. Seubsman, A. Sleigh

**Affiliations:** ^a^ National Centre for Epidemiology and Population Health, Australian National University, Canberra, Australia; ^b^ School of Human Ecology, Sukhothai Thammathirat Open University, Nonthaburi, Thailand

**Keywords:** Thailand, trust, food retail, supermarket, fresh market

## Abstract

Thailand has experienced dramatic growth of large national and international modern food retailers, such as supermarkets, hypermarkets and convenience stores in large cities and regional centres in the last two decades. Nevertheless, Thai consumers continue to purchase perishables (fruits, vegetables and animal products) from fresh markets (wet markets, talat sot) contradicting predictions from analysts that modern food retail chains will rapidly replace fresh markets as the preferred venue for purchasing all types of foods. This paper examines trust in food retail systems as an under-explored dimension lying behind the continued patronage by Thais of fresh markets to purchase perishable items. It derives from a research program commenced in 2005 that includes fieldwork visits, interviews and questionnaires. In the context of the Thai food retail transition, we propose that trust affects relationships between consumers and (1) individual fresh market-based vendors, (2) the food products sold at fresh markets and (3) the food retail system more broadly. If fresh markets can be maintained in the face of sustained pressure from modern national and international food retailers, Thais will continue to use them. Meanwhile, trust is a relatively unrecognised dimension that is supporting the continued existence of traditional food retail formats.

## The Food Retail Transition in Thailand

This paper examines trust in food retail systems as an under-explored dimension lying behind the continued patronage by Thais of fresh markets as a source of perishable items despite the fact that Thailand has experienced a spectacular growth of large national and international modern food retailers. Supermarkets, hypermarkets and convenience stores have spread rapidly from the large cities to regional centres in the last two decades. This dramatic supermarket growth in Thailand, as well as South and East Asia, has more generally been labelled a ‘revolution’ (Reardon, Timmer, and Minten 2012), with analysts observing that it has outstripped western and South American patterns of supermarket growth. This modern retail spread has been driven by economic development, increasingly liberalised foreign investment laws, urbanisation and female workforce participation, among other factors (Reardon, Timmer, and Minten 2012). Retail change is one potential driver of the so-called nutrition transition, which postulates that as countries develop economically and undergo related social and cultural changes, such as those listed above, the population’s diet increasingly shifts to unhealthy, calorie-dense foods, including meats, oils, fats, sugars and processed foods. Modern food retail contributes to this process by making processed, energy-dense foods affordable and more widely available (Hawkes 2008). The nutrition transition contributes to increasing prevalence of non-communicable diseases, such as diabetes and cardio-vascular diseases, which are replacing infectious diseases often associated with under-nutrition as major contributors to mortality and morbidity (Popkin 2001, 2003). Thailand, too, is experiencing a nutrition transition with rising consumption of calorie-dense foods (Kosulwat 2002) and increasing incidence of overweight and obesity (Aekplakorn et al. 2004; Aekplakorn 2011).

Despite the rapid growth and spread of supermarkets in Thailand, many Thai consumers continue to purchase perishables – fruits, vegetables and animal products – mainly from fresh markets, while processed and packaged foods are purchased mainly from supermarkets. This pattern of behaviour supports the contention that a bifurcated retail system is developing in the country (Kelly et al. 2014). This bifurcation, to some extent, expresses a financial divide in which poorer Thai consumers on low incomes are more likely to purchase fresh products from fresh markets, while wealthier consumers purchase most of their food from modern retail formats where the quality of perishables may be better, but prices are higher (Schipmann and Qaim 2011a).

It is not yet clear whether modern food retailers will eventually overtake the sale of fresh produce from fresh markets, particularly in Asian settings (Goldman, Ramaswami, and Krider 2002; Endo 2013). Research in Hong Kong over a four-year period showed shoppers continuing to purchase perishables at fresh markets despite the presence of supermarkets (Goldman, Ramaswami, and Krider 2002). More recently, the notion of a completed ‘distribution revolution’ in Thailand has been questioned (Endo 2013). Nevertheless, Reardon, Timmer, and Minten (2012, 12334–12335) contend that modern retail in South Asia has rapidly gained a market share, including in fruits and vegetables (20–60% in various countries) and that modern retail formats will eventually dominate.

While price, convenience, access and other practical considerations undoubtedly influence consumers’ choice of food retail format, we wish to understand the contribution of an underlying and under-recognised dimension of this behaviour: trust. Much of the debate regarding the extent of the retail ‘revolution’ centres on relative market shares between the competing retail formats, the demographic profiles of the shoppers and the types of nutritional value (fresh versus processed foods) to which shoppers are exposed or have access. Research, thus far, on the success and speed of the supermarket revolution in Asia has highlighted technological developments and strategies such as the modernisation of procurement strategies and investment in infrastructure (Reardon, Henson, and Berdegue 2007; Endo 2013).

In contrast, in Western countries where the mainstream dominance of supermarkets is undisputed, researchers are now turning their attention to sociocultural forces and consumer values to explain the growth of farmers’ markets and other types of direct sales between producers and consumers (Hinrichs 2000). These are cited as examples of alternative food networks (AFNs) which have developed in response to modern, industrial food retail. Granovetter’s (1985) proposal that social relationships, social ties, social capital and trust are embedded, but under-acknowledged, in economic exchanges in modern economic systems has been used to explain the growing popularity of direct agricultural markets (*i.e.* farmers’ markets) as an exemplar of AFNs in advanced industrial countries (Hinrichs 2000).

## Trust

Trust is defined as ‘[c]onfidence in or reliance on some quality or attribute of a person or thing, or the truth of a statement’ (OED online 2015) and is considered a vital element of embeddedness, as described above. In addition, trust has a performative aspect so that discussions of trust often include this latter element. Offer, an economic historian, observes that ‘[t]rust itself resembles a gift; a unilateral transfer with the expectation, but no certainty of reciprocity’. He connects it to ‘regard’, which is an ‘attitude of approbation’, because regard is an ‘incentive for trust’. Trust develops between people and groups over time; if it is not quickly established, the relationship is unlikely to be maintained particularly in the marketplace where it has functional characteristics. It is reliant on a positive feedback loop; the more that trust is rewarded, the more functional and important it becomes to relationships. ‘[It] is efficient: it economises on the “transaction costs” of monitoring, compliance and enforcement’ (Offer 1997, 454). It helps to establish and replicate both social and economic relations. For example, Geertz, describing bazaar economics in Morocco, refers to ‘[c]lientization [which] is the tendency, marked in Sefrou, for repetitive purchasers of particular goods and services to establish continuing relationships with particular purveyors of them’ (Geertz 1978, 30). This practice limits the time and effort required to find a suitable trading partner ‘in whom one has faith’ (Geertz 1978, 31). An alternative form of trust is based, not on the development of a relationship, but instead on a predefined relationship such as kinship, even before a personal relationship has been established. The close relationship between trust and social ties is used to explain, for example, the development of tightly knit, ethnically homogenous middlemen in the marketing of smallholder rubber in Malaysia (Landa 1981). The strategy of trading with ethnically similar people or ‘insiders’ who share a code of ethics reduces the costs of gathering information about suitable trading partners (Landa 1981, 358).

Exploration of the relationship between trust and social relations has been extended to modern nations through increasing interest, over the last three decades, in the role of social capital. The term has been used widely but in differing ways. Putnam, Leonardi, and Nanetti (1993, 167) define it as having ‘ …  features of social organisation, such as trust, norms and networks that can improve the efficiency of society by facilitating coordinated actions’. Others refer to the ways in which institutions, structures and information channels expedite collective action (Coleman 1990), to people cooperating to achieve common goals based on shared norms or values (Fukiyama 2002, 26), or to the resources invested in social networks (Lin 2001). Social capital is theoretically and empirically associated with structures, social norms and reciprocity with trust considered to be a central element. The social capital literature highlights the importance of trust to a range of socio-economic activities and institutions extending beyond close-knit, ethnic or kin-based groups that are required to drive the transition to economically developed modern nations (Fukiyama 2002, 25).

Despite this latter point, social capital and trust are being invoked currently to explain the growth of alternative market forms in modernised, economically developed nations. As we have noted, the concept of trust is being increasingly interrogated for its role in the growth of European and American alternative fresh food retail formats (Sage 2007). This literature is relevant to Thailand, with Thai fresh markets sharing some characteristics with AFNs, such as short food supply chains (Sage 2007) and the importance of trust-based, face-to-face interactions between vendors and purchasers. However, Thai fresh markets do not neatly fit with discourses about AFNs that reflect the very different history and context of the Global North. For example, Thai fresh markets have not emerged as a radical movement reacting to a globalising, industrialised food system that is already fully established (Goodwin 2003). Instead, they represent the maintenance of traditional food networks that operate in economically developing countries. Of relevance to both settings is the need to be cautious about an overly sentimental view of trust, with some noting that economic goals and self-interest remain important for vendors and purchasers (Hinrichs 2000).

With regard to analysing the role of trust in the food retail transition in Thailand, we propose that trust can be placed by consumers in three interrelated areas: (1) individual vendors; (2) the food products they sell and (3) the food retail system more broadly (*i.e.* traditional fresh markets and modern supermarkets). While trust is difficult to disentangle from other elements of embededdness, it is our main focus here. Before examining the concept at the local and institutional levels, we propose that trust in food retail formats cannot be separated from cultural understandings of the concept at a national level. It has been noted that ‘[c]ulturally embedded values, norms and codes of communication are all examples of contextual elements that are likely to influence the way trust emerges in food-related practices’ (Poppe and Kjearnes 2003, 7). Our categorisation of three levels of trust is based on the work of Poppe and Kjaernes, who identified the following levels at which food-related trust operates in Europe: (1) the broad national scale; (2) the individual level of socialised routines and behaviours and (3) the level of institutional arrangements, with all three interacting. In this paper, we use existing literature to examine trust at the national level, which then provides the context in which we investigate the way that trust operates at the other two levels. The second level aligns with trust in individual vendors and their products, and the third level with the institutions of the food retail system represented by supermarkets and fresh markets.

The literature on trust differentiates between generalised trust as ‘belief in the benevolence of human nature in general’ (Yamagishi and Yamagishi 1994, 132) and trust in specific people or things known as knowledge-based trust, or otherwise described as informal social ties (Glanville, Andersson, and Paxton 2013). Consumers’ preferences for generalised trust in modern anonymous food production systems or knowledge-based trust in social relationships and face-to-face interactions are likely to be influenced by national and cultural perspectives on trust, as Poppe and Kjaernes (2003, 7) have observed. Research from other countries suggests that the transition to modern retail formats, such as supermarkets, as well as increasing levels of generalised trust, is characteristic of economically developed, modern societies (Fukuyama 2002).

Thus far, the concept of trust has received little attention in relation to traditional fresh food markets that are being placed under commercial pressure by rapid supermarket growth in the modernising Asian context. Using the framework delineated above, we discuss the limited literature on trust in Thailand and its relevance to consumers’ preferences for food retail formats. In particular, we interrogate the role trust plays in maintaining support for fresh markets which supply many people with fresh, affordable, healthy produce (Banwell et al. 2013) that is appropriate to Thai culinary culture. We address consumer perceptions of modern and traditional retail formats and their representations. These include supermarkets’ self-promotion as purveyors of safe, high-quality food and in clean, modern, environments, in contrast to fresh markets, which are perceived sometimes as dirty, unhealthy and old fashioned. The relevance of Thai concerns with food safety, pesticide use and bacterial contamination is examined (Posri, Shankar, and Chadbunchachai 2006; Roitner-Schobesberger et al. 2008) in the context of trust in food retail systems.

Generalised trust has been interrogated at the national level across 51 countries using the following question from the World Values Survey (Delhey, Newton, and Welzel 2011, 786): ‘Generally speaking, would you say that most people can be trusted or that you need to be very careful in dealing with people?’ In response to the question, national data for Thailand indicate that it has a reasonably high indication of trust in comparison with other countries (Delhey, Newton, and Welzel 2011, 791). A refinement of this question has been adopted to investigate what is meant by ‘most people’ to determine a radius of trust or the ‘circle of people among whom cooperative norms are operative’ (Delhey, Newton, and Welzel 2011, 787). The authors differentiate between an in-group (family and neighbourhood) and an out-group (unknown people, or those of another religion or nationality) and between levels of trust indicating the intensity of cooperation. When the meaning of ‘most people’ is explored among the same 51 countries, Thailand is shown to have a low trust radius, meaning that trust in most people is limited to a familiar in-group in contrast to Switzerland, for example, where the category ‘most people’ is likely to include an out-group. Where trust in an out-group is high, the radius of trust is large, meaning that a wider circle of people is included in the trusted category of most people. The authors observe on the basis of these national comparisons that generalised trust in Asian societies, including Thailand, is low and that, in general, ‘Confucian and less affluent countries appear to have less general trust than the standard question suggests’ (Delhey, Newton, and Welzel 2011, 800). Explanations for this include the reliance on ‘familism’ or the strong family bonds common in Chinese parts of Asia (Fukiyama 2002, 28) and lack of trust in autocratic governments. Thai people are more likely to place trust in familiar social relationships rather than in anonymous actors. Such a finding provides context for our argument.

## Methodology

This paper is based on a 10-year program of research, with two main components:

Face-to-face qualitative interviews have been conducted by the authors with 27 shoppers from 2 fresh markets to explore their perceptions of food availability and social interactions, and a further set of in-depth interviews and observational studies were conducted over a number of years in markets in the 4 major regions of Thailand. These fieldwork sites are described in Table 1. The methodological approach has been described in detail elsewhere (Banwell et al. 2013). In-depth interviews were conducted also with supermarket shoppers in Chiang Mai (Isaacs, Dixon, and Banwell 2010). These qualitative data are used to explore the issue of trust.

**Table 1.** Fieldwork sites.

Region	Major city	Markets
Central	Bangkok	Nonthaburi Provincial Market on Bangkok periphery Sam Chuck Market, 90 kms from Bangkok
Northern	Chiang Mai	Central Market Tanin Market
North Eastern	Khon Kaen	Bang Lam Poo Market
Southern	Nakhon Sri Thammarat	Kukwang Municipal Market Mae Som Jit Market

In addition, we draw upon data from the Thai Cohort Study (TCS), which commenced in 2005. In it, 87,134 out of 200,000 students enrolled at Sukhothai Thammathirat Open University (STOU), a provider of distance education, returned a self-completion questionnaire covering a range of health outcomes and health risks associated with the health-risk transition in Thailand (Sleigh et al. 2007). A different, self-completion questionnaire was sent to a sub-sample of 3400 members of the TCS in 2012 (Kelly et al. 2014, 447–449). They were selected from the TCS database and their postcodes were used to include all cohort members from one urban centre and the surrounding hinterland in each of the four regions of Thailand. The questionnaire ascertained where consumers shopped, the characteristics of their food retail preferences, food provisioning and diets. Participants were asked whether they had access to traditional food outlets (fresh market, general store or mobile vendor) and or modern outlets (convenience store or supermarket). Based on information about the frequency with which they purchased food at these outlets, they were classified as people who mainly shopped at traditional, modern or a mix of retail formats. We obtained responses from 1516 sub-sample members (45%), and in-depth interviews were conducted with a further subset of 16 respondents to explore the survey responses in detail. Descriptions of cohort recruitment and study design have been published in detail (Sleigh et al. 2007; Kelly et al. 2014).

## Consumers’ Food Purchasing Patterns

The 2012 survey of a TCS sub-sample of 1516 respondents provided an overview of food retail use. More respondents were female (56%) and urban dwelling (75%). More than 80% of them had access to both traditional and modern retail formats, with some categorising themselves as mainly traditional (fresh market) shoppers (53%), as people who shopped at both (mixed shoppers) (18.9%) or as mainly supermarket/convenience store (modern) shoppers (27.9%) (Kelly et al. 2014, 451). Rural dwellers and those who had recently moved to urban centres were more like to purchase food at fresh markets, as were older people, those with lower incomes and those living in larger households. Residents in Northern Thailand were more likely to shop at fresh markets than those living in Bangkok or the Central Region. However, most respondents purchased fresh meat (80%) and vegetables and fruits (86%) at fresh markets (Kelly et al. 2014, 452). In the survey, questions were asked about the everyday and concrete store characteristics that influenced shoppers’ retail choices. Hygiene or cleanliness was rated highly, followed by the variety of food on sale, and the convenience of the store location. Supermarket shoppers were slightly more likely than fresh market shoppers to rate hygiene highly, although fresh market shoppers also considered it important (Kelly et al. 2015). This raises the question: why do fresh market shoppers who are concerned about hygiene continue to patronise fresh markets rather than the apparently cleaner supermarkets to buy the sorts of foods that can readily transmit disease? One obvious answer is price, which is undoubtedly an important issue for Thai shoppers. Fresh markets generally sell fresh products more cheaply than supermarkets (Schipmann and Qaim 2011a) and in smaller sizes, allowing consumers to pay for the quantity they need rather the quantity designated by the retailer. The relevance of price is supported by the fact that those with higher incomes were more likely to purchase perishables at supermarkets (Kelly et al. 2014).

## Relations Between Fresh Market Vendors and Consumers

Food is imbued with many characteristics related to taste, to cultural valuing and to convenience, among others, which consumers take into account as well as price. This research points to the role of trust among this constellation of valued attributes. During market-based ethnographic research, we gathered comments from participants that suggested that their choice of retail site was not about a choice between a cheaper fresh market or a more expensive supermarket, but instead signified a preference for a particular fresh market. One respondent indicated purchasing perishables ‘only at this market’ (Shopper at Nakorn Sri Thammarat). This preference for a particular market to buy fresh food indicates that the market meets an additional need beyond price.

Consumers said that their choice of a particular fresh market was influenced, in part, by their relationships with specific vendors, as well as their purchasing habits. Market-based fieldwork illustrated the complex ways in which fresh markets are integrated into shoppers’ daily rituals and thus anchor a way of life for many consumers and for vendors. Consumers and market vendors constitute a social network through consumers incorporating market visits into their daily routines, such as shopping on their way home from work or other activities.

One informant likened the relationship between consumers and vendors to one between siblings: ‘We bring a brother–sister (*phi-nong*) relationship between buyers and sellers … it’s still present, but not as much as the past’*.* This statement illustrates the closeness of the relationship and the integration of it into the norms of Thai culture. In Thai society generally, non-kin relationships are often expressed as kin relationships. For example, the elder–junior sibling relationship provides a model for social conduct and may reflect power relationships and emotional ties (Howard 2007). Not only does the use of kin terms denote a close connection, but it also implies a hierarchical relationship of ‘mutual dependency’ (Howard 2007, 207). Indeed, the ‘sibling relationship … is the kin relation most commonly invoked as a model for non-kin relationships’ (Howard 2007, 208). The repeated contact and close relationship between buyers and sellers are consolidated and expressed by the use of sibling kin terms.

A shopper drew attention to the importance of the relationship with vendors, saying: ‘People of my age love the relationship with the sellers’*.* This reference to the shopper’s age as an influential factor in the relationship was reiterated by other respondents who observed that a close interpersonal association was more valued by older shoppers. It is plausible that fresh market shoppers were more likely to be older or retired with more time to invest in relationships. However, analysis of the 2012 survey data on retail choice by age did not show a statistically significant difference. Over 84% of participants aged over 45 and 87% of those under 45 purchased fruit and vegetables at a fresh market (Kelly et al. 2014, 451).

Trust in the relationship overflowed into the financial transactions between buyers and sellers. An older, low-income woman from Chiang Mai observed: ‘Fresh market stall holders look after their customers more than the supermarket. They try not to put up their prices too much.’ She thus implied that a social relationship trumps a purely economic exchange. One could argue that a vendor’s apparent generosity reflects a canny economic decision to invest in a long-term relationship that brings the customer back repeatedly. Nevertheless, trust is still required by the shopper in judging that the prices are indeed reasonable and by the vendor in endeavouring that the shopper will return; in other words, a relationship of delayed reciprocity is established. Another shopper said that when close relationships existed, vendors would reduce prices or provide credit. Shoppers also noted that they trusted fresh market vendors to give the best prices when produce was cheaper because it was in season while supermarkets were not flexible on price. As one person commented, ‘At the supermarket you just have to accept the price and range that they have’*.* In other words, there was no ability at the supermarket for vendors and shoppers to negotiate a price based on their relationship.

## Relations Between Supermarkets and Consumers

Participants during interviews described a lack of personal relationships with supermarket staff. This was seen as a positive factor by some whose comments included: ‘The staff are always polite at the supermarket, they help you if you need it’. For most, however, the service at the supermarket was seen as impersonal: ‘people at the supermarket help you if you ask, but they generally just leave you alone otherwise’. Thus, supermarkets offer service consisting of polite, impersonal and deferential behaviours by staff to shoppers (Isaacs, Dixon, and Banwell 2010), instead of personal relationships, to assist them to purchase goods more easily. Participants purchased snacks, desserts, packaged or processed foods, ready-to-eat or dairy foods rather than fresh foods, from a range of retail formats, including supermarkets. A young man from Bangkok who earned a high income said: ‘I like that I can go to the fresh market and buy my traditional fruit and vegetables and then go to the supermarket for modern foods.’

## Perishable Products

It appears that trust in a familiar and positive relationship can be transferred to the product itself. The close social proximity which builds trust between the vendor and buyer is replicated in the spatial proximity between the product and the shopper and also builds trust. Thus, fresh market products reflected close ties between vendors and shoppers and were also valued because they were ‘Thai vegetables’ or ‘local food from home gardens’. Thais who shopped at fresh markets often said that they did so because they valued local produce. Shoppers could also enquire about the source of the produce from the vendor; because they trusted the vendors, they trusted their responses about the product. Symbolically, as well as spatially, the two are related; geographical proximity provides a source of information about how the product is produced and how far it has travelled, both qualities relevant to food safety and freshness.

The ideal of the local encapsulated the close relationship between the actors and product. For example, vendors at two fresh markets in Chiang Mai said that they bought fresh produce from either local producers or the local wholesale market. At one market, nearby hill tribe members brought fresh food into the market to sell every morning, while some stall holders also grew their own products for sale. Fresh market shoppers reported that they thought that most of the produce they bought was ‘local’ or came from ‘around here’ or from nearby provinces. Fresh markets also sell food products that are certified as deriving from a specific geographic area under the Geographical Indications Protection Act (2003). Consumers were also aware that some products sold at fresh markets were not grown in Thailand, but they could easily ask vendors about this, or it was indicated on signs placed in the stalls.

The relationship between proximity and trust was not always clear. This was exemplified by two participants who discussed mobile vendors who sell fresh produce from their vans or in plastic bags carried on motor cycles. One young male resident living in Bangkok said that he did not trust the products because he did not know where the produce came from or how fresh it was. In contrast, an older male participant said that he trusted the produce from mobile vendors because he bought from the same family of vendors who visited him every day. Yet another younger male Bangkok resident shopped from specific market stall holders because he had a personal relationship with them based on their derivation from the same region of Thailand in which he was born.

Considerable media attention and concern have been expressed over the last decade or so about food safety, especially related to pesticide residues on fruit and vegetables. On this matter, consumers’ views diverged; some considered fresh market produce to be less likely to be contaminated by pesticides or to be unhygienic, while others thought that supermarket foods were safer and of better quality. Fresh market shoppers were more likely to consider the locality of the food and its appearance rather than labels and certification when judging food safety and pesticide contamination. An older male resident of Bangkok with a low income stated:

I don’t like the idea of pesticides on fresh food, but I don’t believe any of the foods labelled ‘organic’ are any different. I don’t trust any of those certification labels, like ‘Doctor’, ‘Clean and Healthy’ etc.

In contrast, supermarket shoppers placed more faith in the labels and certification that was used on supermarket products declaring them to be hygienic, organic or safe.

Produce bearing the label of the Royal Project derived additional status relative to other labels due to its association with the trusted Thai Royal Family and the King in particular. Large formal portraits of members of the Royal Family are common in public spaces in Thailand, rendering them familiar and trusted, yet socially distant due to their superior status. In addition, Thais are familiar with the history and purpose of the Royal Project, which was established by King Bhumibol Adulyadej in 1969 to provide alternative cash crops for poor hill tribes farmers so that they could avoid deforestation or opium production. This sought-after produce is sold mainly at fresh markets and to a lesser extent in supermarkets, as this person suggests.

I worry a lot about pesticides in my food. I like to buy the certified clean vegetables, like the Doctor Brand. They sell them at my local fresh market. I trust the Royal Project the most, though. They have a special stall at a weekend-only night market in my area, and I try to make a special effort to go there.

The cleanliness of the environment and the use of packaging convinced some patrons that supermarket food was safe. For example, a Chiang Mai consumer said that she preferred bread to rice because it was a more a more sanitised food (Isaacs, Dixon, and Banwell 2010). In contrast, a young woman from Bangkok said, ‘I would never buy fresh food from the supermarket. The quality and freshness is not good enough’.

## Food Retail Institutions

Our survey results, reported above, showed that most Thais preferred to buy fresh produce from fresh markets. Hygiene or cleanliness was rated most highly in influencing choice of food retail format (Kelly et al. 2015), but it was more important to supermarket shoppers than fresh market shoppers. Based on ethnographic data, it appears that participants regarded the produce from fresh markets as fresher because it is locally sourced and spends less time being transported and stored, while supermarket environments were deemed more hygienic. Although closely related, these concepts illustrated differences between the two formats. Hygiene is about cleanliness and control of the spread of infectious disease, while freshness refers to the time taken from when a product is harvested and sold to the consumer; both have implications for food-borne disease. In Thailand, refrigerated storage and transporting facilities are relatively new and are used mainly by supermarket supply chains, while fresh market supply chains are generally unrefrigerated. Supermarkets use modern supply chains and techniques (*e.g.* Hazard Analysis Critical Control Point) for identifying and reducing the transmission of food-related infectious disease. In the process, they extend the time period during which food can be stored, purchased and safely eaten.

Over the last two decades, more Thai households have begun to acquire fridges, which are often small and sometimes without a freezer, thus limiting storage of perishable food. With little capacity to store food safely for a long period, Thais may prefer foods that have not travelled long distances from ‘paddock to plate’. They shop frequently or even daily to prevent food-borne disease. Just as important, however, is a strong and long-standing cultural valuing of fresh ingredients (Seubsman et al. 2009). Shoppers judge freshness by flavour, smell and appearance to decide whether a product is suitable for purchase and consumption. These subjective and sensual approaches to foods are buttressed by knowledge of products’ provenance (*i.e.* local) and by previous interactions with the vendor (trust). Thus, supermarkets and fresh markets employ very different procedures for developing trust in food safety: one is impersonal and technologically driven, and the other is personal, based on short food supply chains and interpersonal relations.

While it is acknowledged by Thais that supermarkets have a place in food retailing, supermarkets are sometimes regarded as outsiders and a threat to local communities, particularly when they are located too close to a fresh market. A local trader commented: ‘If it [the international supermarket chain] is far and does not affect the community's economy, I'm okay, but otherwise it could be a threat’.

This outside status locates supermarket chains, especially those owned by international companies, at the opposite end of the social and spatial continuum from fresh markets. Not only do supermarkets place pressure on the economic viability of fresh markets as a whole, but also they potentially threaten the livelihood of the usually female stallholders and wholesalers who often run family businesses. Some Thais appear to weigh up the convenience of supermarkets with their concern for the survival of stall holders who were described as ‘poorer community members’*.* Thais express a desire to have the comforts of a supermarket, while retaining fresh markets for their social value. Fresh markets are attempting to respond by improving their appearance, infrastructure and food safety standards where possible.

To overcome their social and spatial distance from everyday Thais, supermarkets are attempting to replicate Thai-ness and to integrate themselves into Thai cultural life. Their attempt to position themselves as experts and authority figures on food is a strategy that supermarkets have employed in many countries (Dixon 2008). In addition, in Thailand, supermarkets are mimicking fresh markets’ traditional embeddedness in community and religious festivals. We observed in a Southern Thai town how local fresh market stallholders sold special festival foods during an important religious festival. Meanwhile, uniformed staff members from the major supermarket in town participated in the festival parade, holding a large banner advertising the supermarket’s name. Isaacs (2009) observes that, increasingly, supermarkets and hypermarkets are replicating the appearance of fresh markets by establishing small food stalls in their forecourts. Such activities attempt to build trust by referencing the elements that constitute trust for Thais, namely familiarity, locality and close proximity, and elements of Thai culture. Nevertheless, supermarkets have difficulty reproducing an authentic version of the close and long-standing relationships between consumers and vendors that are an important element in the continued success of fresh markets in selling perishable food items.

## Discussion

In this paper, we aim to explore the nature of the transition from traditional to modern food markets in Thailand. We argue that the transition, which has health and social consequences, is occurring, but not as rapidly as expected. This paper draws on different types of data to propose that trust is an under-acknowledged dimension that contributes to Thai adherence to fresh markets in the face of sustained efforts by supermarkets to win their loyalty. The little research that has been conducted in Thailand on fresh markets and food retail has focussed mainly on issues related to price and affordability and the ability of large supermarket chains to distribute food efficiently. Our survey data show that despite modern supermarkets’ ‘proactive fast-tracking strategies’ to enable their rapid entry and growth into developing countries (Reardon, Henson, and Berdegue 2007), a majority of Thais of all ages continue to purchase fresh produce from fresh markets. Most survey respondents aged in the 36–50-year-old group were as likely to shop in fresh markets as supermarkets (Kelly et al. 2014), suggesting that younger Thais will retain their allegiance to fresh markets.

Interview-based data indicate that while convenience, price and other material factors play a major role in the consumers’ purchases of packaged goods from supermarkets, personal, knowledge-based trust is also a consideration when Thais purchase fresh produce. A trusting relationship is embedded in everyday social and commercial transactions. Fresh market shoppers were likely to build relationships with their preferred traders and establish social networks based on familiarity and mutual benefit in a way that shows similarity to Kirwan’s (2006) and Offer’s (1997) concept of regard. The trust that Thai shoppers develop in their social relationships with fresh market vendors spilled over to the foods that were traded, and, to a lesser extent, the institution itself, while offering at the same time an efficient way of undertaking food purchases (Offer 1997). These relationships referenced concepts of the local, freshness and Thai-ness, all of which draw on social and spatial proximity, as well as Thai culture, tradition and continuation of past practices. In this regard, there are some similarities with AFNs in the Global North in valuing freshness, the local and connecting to an existing culinary culture. Another shared characteristic is short food supply chains which consist of either face-to-face interactions between producers and consumers, or ‘relations of proximity’ (Renting, Marsden, and Banks 2003, 400) that include produce grown in the region, or cultural proximity where produce references local cultural attributes. The similarities are not sustained, however. For example, produce from Thai fresh markets does not draw heavily upon ‘ecological characteristics’ such as organic or non-genetically modified produce in the same way that AFNs do (Renting, Marsden, and Banks 2003, 400), although there are some moves in this direction.

The Thai preference for buying perishable foods at fresh markets also displays many of the elements of regard described by Offer (1997). Referencing the great ethnographic accounts of gift exchange, such as the *potlatch* ceremony by Mauss and Malinowski’s *Kula* ring, Offer (1997, 451) notes that through these exchanges, reciprocity is expected, although it is often delayed and the value of the reciprocated gift is often unspecified. Offer (1997, 452) argues that ‘exchange is not only an economic transaction, it is also a good in itself, a “process benefit” usually in the form of a *personal* relationship’. He lists a range of valued personal attributes that are relevant to vendors and purchasers, including respect, acceptance, reputation, status, power, friendship, kinship and sociability, under the term ‘regard’. A gift signals regard, and as we have already noted, trust is a type of gift (Offer 1997, 454). In Thai fresh markets, trust and mutual regard between vendors and shoppers facilitate long-standing exchange relations that are signalled and supported by transactions that have some gift-like characteristics, such as a reduced price, with shoppers reciprocating by continuing to patronise their favoured vendors. Trust is integral to the close relationships, as well as to local, fresh foods and ingredients used in Thai foods that are found at fresh markets, with their embedded social relations that build upon Thai culture, spirituality and cultural practices. Additional evidence to support the contention that knowledge-based, face-to-face trust is important in food-related economic relations in Thailand is that Thai small farmers do not like to sell their produce based on market exchange. For example, Thai sweet pepper farmers prefer ‘marketing options that do not involve a contract’ (Schipmann and Qaim 2011b, 667). Schipmann and Qaim concluded:

remarkably, the most important factor is the relationship between farmers and buyers. The positive utility associated with knowing the buyer personally seems to outweigh the negative utility associated with entering into a contract in general, which is probably related to issues of trust. (2011b, 676)

Regional and cultural differences abound in Thailand. However, our survey data did not show clear differences among the four regions in terms of choice between types of food retail outlets. Bangkok consumers were more likely to be mainly supermarket shoppers (36%) than people from other regions (Kelly et al. 2015, 451), although this finding may reflect the fact of their greater access to and familiarity with, and acceptance of, supermarkets because they have been operating in the capital for longer than elsewhere in the country. From our survey data, it is difficult to disentangle cultural differences across regions from other issues such as convenience, accessibility and availability of food retail forms. However, local cultural differences, which were reflected in consumers’ purchasing patterns, could be seen in dietary preferences, the purchase of particular locally grown products and the sale of products suited to local culinary styles. Across the country, women were central in fresh markets as stall holders and sometimes owners and were valued by shoppers. One of the reasons that Thai consumers gave for supporting fresh markets was that they offered a livelihood for these women who would be unlikely to find alternative forms of employment.

It is likely in Thailand, as in countries such as Portugal and Spain, where trust is knowledge-based rather than generalised, that there is less likely to be trust in the food system in general (Poppe and Kjearnes 2003). Isaacs, Dixon, and Banwell (2010) argue that in contrast to fresh markets, supermarkets generate ‘standardized perfection’, employing impersonal interchanges with sales people, while attempting to simulate some of the social, community and spiritual activities of fresh markets. It could be argued that these community activities are ‘gift-like’ and may be aimed at generating a reciprocal relationship with consumers. However, despite supermarkets’ attempts to borrow and replicate trust, they do not appear to be succeeding, at least in the short term, without the central ingredient of close social relationships. Thais’ trust in them is unlikely to grow markedly, while there is still a marked cultural, social and spatial distance from them.

It is conceivable that, in the future, some major trends will assist supermarkets to gain ascendance: one is supermarkets’ ability to compete on price, supported by hygiene, convenience and comfort. In the case of the latter trend, supermarkets in Western countries have been able to undercut traditional food retailers, such as street store greengrocers and butchers, on convenience and price, thus driving them from the market place. They have continued to compete among themselves on the basis of low prices (Dixon and Banwell 2016), although once they are have market dominance, the price of perishable goods increases. In Thailand, supermarkets attract customers on the basis of lower prices for some fresh produce, although they are more expensive overall than fresh markets where poorer Thais still buy cheaper, but sometimes lower quality fresh goods, and in smaller quantities that suit their budgets and storage capacity. The large fresh markets that operate as distribution centres appear to be able to resist urban development but the owners of small to medium-sized markets have been known to sell their land to real estate companies who replace them with high-rise buildings. The growth in motorised transport and roads may also impact. However, informal fresh markets often appear in convenient locations but are not able to sell wet products (raw meat and fish).

Another trend is the shift from face-to-face trust, as Thailand modernises, to generalised trust that accompanies the transition to modernisation. Trust itself is a complex and elusive concept (Yamagishi and Yamagishi 1994) which could be explored in greater depth. Generalised or out-group trust is lower in Asian than in Western societies and is especially low ‘[in] South Korea, Thailand’ (Delhey, Newton, and Welzel 2011, 800). The role of Confucianism has been cited as a reason for low generalised trust and high face-to-face trust in countries like China. Thailand is Buddhist, with 94% of the TCS sample identifying as such (Sleigh et al. 2007). However, the high proportion of Thais with Chinese ancestry may have contributed to a cultural valuing of in-group trust in Thailand that is similar to China’s reliance on kin-based trust (Fukuyama 2002). The influence of the small proportion of Moslems in the South of Thailand is not apparent in our research; in general, reports on trust in Islamic countries vary (Delhey, Newton, and Welzel 2011; Delhey and Welzel 2012). Bangkok has slightly higher levels of generalised trust than in other urban or rural areas (Yiengprugsawn et al. 2011), which, with its higher use of supermarkets, suggests a more modern approach to economic exchanges. It has been argued that the growth of out-group trust has stronger links to economic, political and modernisation than religion (Delhey and Welzel 2012).

However, the growth of social capital and the development of more generalised trust in Thailand may be impeded by accusations of government and business corruption that has spread to the food retail industry. A recent media report suggests that a major food producer and retailer have been making regular payments to media outlets, although the company denied that it was buying favourable publicity (*Bangkok Post* 2014). Two other macro-level trends may have implications for the role of trust in food retail. One is the increasing numbers of mainly Burmese migrants now living in Thailand (Martin 2007) who are said to be gaining work in Thai fresh markets. It is likely that this too will undermine in-group trust in the future, if it continues and may contribute to a shift towards supermarket shopping. Another is the military coup in May 2014, which may have had an impact on the development of social capital that is associated with democratic growth (Fukuyama 2002).

## Conclusion

Ultimately, the centrality of knowledge-based trust to the purchase of perishable foods has played a somewhat unrecognised role in maintaining the viability of fresh markets, as supermarkets have made considerable efforts to gain market share in Thailand. Economists, and others, who believe that supermarkets will inevitably take over food retail due to their technological and marketing strengths have been surprised at the tenacity of fresh markets (Reardon, Timmer, and Minten 2012). We argue that for reasons of population health, among others, fresh markets need protection from the power and wealth of the major national and international food retail companies. Policy-makers need to encourage the preservation of fresh markets for the sake of the livelihoods of the stall holders, the preservation of a Thai culinary culture and way of life, and the health benefits of the affordable perishable foods that fresh markets provide.

This paper describes contradictory trends relating to the role of trust in food retail. The growth of generalised trust, social capital and democracy are associated with national economic growth in which national and international supermarket chains play a part. However, we are proposing that the preservation of fresh markets in the face of supermarket growth will protect food affordability for poor Thais and assist them to conserve a healthy, culturally appropriate diet. In part, fresh markets rely on a face-to-face, knowledge-based trust – a form of trust which is generally superseded over time by the more modern form of generalised trust that can be found in Western or developed countries. We are arguing therefore for the value of a form of trust and social capital that could be seen by some as outmoded, and associated with undemocratic institutions. However, countries are complex with uneven patterns of cultural, social and economic change. Even in Western developed nations with high levels of general trust, there has been a substantial growth in AFNs that incorporate small-group social capital and face-to-face trust. These networks are designed to provide local, healthy and sustainable food in response to the loss of trust experienced by consumers in the wake of food scares associated with a globalised, industrial food system. Can Thailand leapfrog the trend to establish new AFNs, by safeguarding their fresh markets without threatening national economic or democratic development? We believe that it can with support from urban, regional and national bodies.

There is a further dimension to the debate and that concerns consumer views on whether the retail transition with which they are wrestling is positive or negative: for their health, for their sense of national identity and modern citizenship, for their support for local livelihoods and so on. This debate concerns not only what is currently happening in food retail throughout Southeast Asia, but also what should happen to it in the future. In other words, what sort of food retail system do Thais want? Modern, large national and international food retail chains in Thailand exemplify a middle-income country’s embrace of global economic development, while traditional food retail formats could be seen as a manifestation of local social systems.
